# Symptoms to Use for Diagnostic Criteria of Hwa-Byung, an Anger Syndrome

**DOI:** 10.4306/pi.2009.6.1.7

**Published:** 2009-03-31

**Authors:** Sung Kil Min, Shin-Young Suh, Ki-Jun Song

**Affiliations:** 1 Department of Psychiatry, Yonsei University College of Medicine, Seoul, Korea.; 2 Department of Psychiatry, Pochon CHA Medical School, Seongnam, Korea.; 3 Department of Biostatistics, Yonsei University College of Medicine, Seoul, Korea.

**Keywords:** Hwa-byung, Symptoms, Anger syndrome, Anger, Depression

## Abstract

**Objective:**

The aim of this study was to identify the characteristic symptoms which can be used for the diagnosis of hwa-byung, a culture-related anger syndrome in Korea.

**Methods:**

The symptoms of the Hwa-byung Scale were correlated with the Korean versions of the Hamilton Depression Rating Scale (K-HDRS) and the State and Trait Anger Inventory (K-STAXI) in 89 patients, who were diagnosed as having major depressive disorder, dysthymic disorder, anxiety disorders, somatoform disorders, or adjustment disorder according to the Diagnostic and Statistical Manual of Mental Disorders, fourth edition (DSM-IV) criteria and who had self-labeled hwa-byung. Also, the symptoms of the Hwa-byung Scale were correlated with each other.

**Results:**

The symptoms of the Hwa-byung Scale which were significantly correlated with the state anger of the K-STAXI but not with the depressive mood (item 1 of K-HDRS) included feelings of unfairness, subjective anger, external anger, heat sensation, pushing-up in the chest, dry mouth, and sighing. The symptoms which were significantly correlated with state anger and depressed mood included respiratory stuffiness, "haan" and hate. The symptoms which were not significantly correlated with depressed mood and state anger included going-out, epigastric mass, palpitation, headache/pain, frightening easily, many thoughts, and much pleading. These symptoms also showed higher correlation with each other in the correlation matrix.

**Conclusion:**

Our findings suggest that hwa-byung is different from depressive syndrome in terms of its symptom profile, and suggest what symptoms should be included in the diagnostic criteria of hwa-byung, an anger disorder.

## Introduction

Hwa-byung (HB), whose literal meaning is "anger disease" or "fire disease", is known as a culture-related syndrome related to anger in Korea^1^,^2^ and is listed in Appendix I, Glossary of Culture-bound Syndrome of Diagnostic and Statistical Manual of Mental Disorders, fourth edition (DSM-IV). Hwa means anger and fire and byung means disease or disorder. HB is known to be a chronic anger syndrome, in which anger is thought to have been chronically suppressed and "accumulated and become dense" and to be characterized by unique symptoms including partially suppressed subjective anger and somatic and behavioral manifestation of partially expressed anger.^3^-^5^ HB is reportedly found in 4.1% of the general population of Korea and is more frequent in middle-aged or older housewives of the lower social class.^6^ Generally, HB is an illness in Korean women who suppress their anger resulting from family conflict, so as not to jeopardize harmonious family or social relationships, because of the confines of traditional Korean culture.^3^,^7^,^8^ Various treatment modalities have been suggested for this culture-related anger syndrome.^9^ Recently, an functional magnetic resonance imaging (fMRI) study suggested that the suppression of anger in HB patients was associated with functional impairment in the anterior cingulated cortex.^10^

When patients with self-labeled HB were diagnosed according to the criteria of DSM-III, many of them were diagnosed as having depressive disorders (major depression or dysthymic disorder), atypical somatization disorder (with Korean culture-related somatic symptoms of HB) and generalized anxiety disorder.^6^,^11^ Therefore, some western psychiatrists consider HB as a culturally patterned way of expression for Koreans experiencing major depression.^12^

This paper describes how the symptom profile of HB differs from that of depression. Any symptom of HB that is significantly associated with anger, but not depression, should be considered as a characteristic symptom to be included in the diagnostic criteria of HB.

## Methods

The subjects in this study were male and female Korean patients 18-65 years of age who visited the department of psychiatry for the first time and were diagnosed as having major depressive disorder, dysthymic disorder, anxiety disorders, somatoform disorders, or adjustment disorder according to the DSM-IV criteria. These inclusion criteria for the diagnosis were made according to previous diagnostic studies on HB.^6^,^11^ The DSM-IV diagnosis was made with the Korean version of Structured Clinical Interview for DSM-IV Axis I Disorders (SCID-I).^13^ Patients who complained only of self-labeled HB were also included. Subjects were excluded if they had major depressive disorder with psychotic features, substance use disorders, mental retardation, clinically significant neurological or physical disorders, were pregnant, had taken any psychotropic drugs during the 2 weeks prior to inclusion in the study, or refused to participate in the research. The research protocol was cleared in advance by the Institutional Review Board of Yonsei University Medical Center. After providing them with a complete description of the study, written informed consent was obtained from all of the participants.

The symptoms of HB were assessed using the Hwa-byung Scale (HB Scale).^14^ This HB Scale consisted of the 22 most common symptoms frequently shown by patients with self-labeled HB^3^,^4^: subjective anger, "uk-wool and boon" (a Korean culture-related sentiment related to social unfairness), external anger, heat sensation, pushing-up in the chest, respiratory stuffiness, going-out, epigastric mass, palpitation, insomnia, headache/pain, dry mouth, anorexia, frightening easily, sighing, sad mood, "haan" (a Korean culture-related sad sentiment related to hard life and social unfairness resulting not only from the tragic collective national history, but also from a traumatic personal life),^15^ many thoughts, hate, anxiety with agitation, guilty feeling, and much pleading.

Each symptom was assessed on a 1 to 5 point scale. Depression in the participants was assessed with the Korean version of the 17-item Hamilton Depression Rating Scale (K-HDRS).^16^

Two psychiatrists (Min and Suh) assessed the participants using the HB Scale and K-HDRS. The inter-rater reliability was significant in the HB Scale (n=27, r=0.94, p<0.0001) and K-HDRS (n=27, r=0.93, p<0.0001).

The participants were asked to rate their anger according to the Korean version of the State and Trait Anger Inventory (K-STAXI),^17^ which includes subcategories of the degree of anger, trait anger, anger-in, anger-out and anger-control.

A correlation analysis using Pearson's correlation coefficients was performed to identify the relationship between each symptom of the HB Scale and the K-HDRS or K-STAXI categories. As the K-HDRS contains some symptoms which may be due to anxiety or somatization, the symptoms of HB were correlated with the score of item 1, depressed mood, and the total score of the K-HDRS as well.

Also, the symptoms of the HB Scale were correlated with each other. Two-tailed tests were used, and p values<0.05 were considered indicative of statistical significance. All statistical tests were performed with Statistical Package for Social Science (SPSS) version 12.0.

## Results

### Sociodemographic data and DSM-IV diagnosis of subjects

Of the 102 participants, 13 patients failed to complete the K-STAXI and, finally, 89 patients were included in the study; 16 males (18.0%) and 73 females (82.0%), with a mean age of 48.03 years (SD=14.38).

The diagnoses of the 89 patients were as follows: major depressive disorder (MD), 45 patients (50.6%); depressive disorder NOS, 3 patients (3.4%); MD and dysthymic disorder (DD), 6 patients (6.7%); MD and generalized anxiety disorder (GAD), 2 patients (2.3%); MD, DD, and GAD in 1 patient; MD, DD, and panic disorders in 1 patient; MD and somatoform disorder NOS in 1 patient; DD in 1 patient; GAD, 4 patients (4.5%); anxiety disorder NOS, 2 patients; GAD and somatoform disorder NOS in 1 patient; posttraumatic stress disorder, 2 patients; panic disorder, 3 patients; obsessive-compulsive disorder, 2 patients; somatoform disorders, 3 patients; somatoform disorders NOS in 1 patient; undifferentiated somatoform disorder, 6 patients (6.7%). Five patients (5.6%) were not diagnosed as having any DSM-IV disorder, but they said they had HB.

### Correlations

Of the 22 symptoms on the HB Scale, those symptoms which were significantly correlated with state anger and/or anger-out but not with depressed mood (item 1 of K-HDRS) included feelings of unfairness, subjective anger, external anger, heat sensation, pushing-up in the chest, dry mouth and sighing (Table 1). Those symptoms which were significantly correlated with state anger and depressed mood included respiratory stuffiness, haan and hate. Those symptoms which were not significantly correlated with depressed mood and state anger included going-out, epigastric mass, palpitation, headache/pain, frightening easily, many thoughts, and much pleading. Those symptoms which were significantly correlated with depressed mood but not with state anger included insomnia, sad mood with tears, anxiety with agitation and guilty feeling. Anorexia was not significantly correlated with depressed mood, but was negatively correlated with trait anger. The total score of the K-HDRS was significantly correlated with all of the HB symptoms, except heat sensation, going-out, epigastric mass, dry mouth, anorexia, frightening easily, and much pleading.

The score of category of anger-out in the K-STAXI was significantly correlated with external anger, heat sensation, pushing-up, sighing, sad mood, and hate, while the score of anger-in category was only significantly correlated with subjective anger and heat sensation. However, no symptoms of HB were significantly correlated with the score of anger-control category. The HB symptoms that were significantly correlated with most categories of the K-STAXI included subjective anger and heat sensation, followed by sighing, hate and pushing-up in the chest. Feelings of unfairness, external anger, respiratory stuffiness, dry mouth and haan were only correlated with the state of anger.

### Correlation matrix

Table 2 shows that subjective anger, feeling of unfairness and heat sensation were more frequently correlated with sighing, pushing-up, sad mood with tears, many thoughts, anxiety with agitation, insomnia, hate, respiratory stuffiness, palpitation, expressed anger, and haan than the other symptoms were.

## Discussion

This study suggests that HB is different from depression in terms of its symptom profile, although they may share some symptoms. This study also suggests that 7 symptoms were significantly correlated with state anger but not with depression, including feelings of unfairness, subjective anger, expressed anger, heat sensation, pushing-up in the chest, dry mouth, and sighing. These symptoms are also included in the group of HB symptoms which was revealed in the correlation matrix and, therefore, should be considered as essential symptoms for the diagnosis of HB. The three symptoms, viz. respiratory stuffiness, haan and hate, which were significantly correlated with state anger and depressed mood as well, should also be considered as characteristic symptoms of HB. These are also included in the group of symptoms which was revealed in the correlation matrix. The seven symptoms (going-out, epigastric mass, palpitation, headache/pain, frightening easily, many thoughts, and much pleading), which were not significantly correlated with depressed mood or the state anger of the K-STAXI, may be considered non-specific but meaningful somatic and behavioral symptoms of HB, because patients with self-labeled hwa-byung frequently complain of them.

Insomnia, sad mood with tears, anxiety with agitation, and guilty feelings, which were significantly correlated with depressed mood but not with state anger, should not be included in the criteria of HB. Anorexia, which was negatively correlated with trait anger, should not be included in the diagnostic criteria of HB either. Headache and frightening easily are common symptoms of depression and anxiety and should be excluded from the diagnostic criteria of HB. Going-out can be considered as a non-specific behavioral symptom and should not be included in the criteria. However, all of these symptoms may remain in the HB Scale for the evaluation of individuals with HB.

Finally, 14 symptoms should be included in the diagnostic criteria of HB, viz. subjective anger, feelings of unfairness (uk-wool/boon), expressed anger (verbal/behavioral expressions of anger), heat sensation, hatred, "haan", pushing-up in the chest, epigastric mass, respiratory stuffiness, palpitation, dry mouth, sigh, many thoughts, and much pleading.

Of course, the symptoms used for the diagnosis of HB should not be simply symptoms of anger such as trait anger or state anger which were highly rated in the STAXI. However, because the authors have conceptualized HB as being the result of chronic suppressed, repeated anger, the tentative symptoms to use for the diagnosis of HB should be tested with an anger rating scale. However, the STAXI was the only rating scale for anger available in Korea and was therefore used in this study. If anger is repressed, somatized or displaced, it might not be manifested as a symptom of the STAXI. Therefore, some symptoms which were not significantly correlated with the K-STAXI, but which are frequently complained of by patients with self-labeled HB, can be included in the HB Scale.

The total K-HDRS score was significantly correlated with most of the symptoms of HB, suggesting that the K-HDRS is inappropriate for differentiating HB, an anger syndrome, from depressive disorders.

There were some limitations to this study. One was the small number of subjects. Nevertheless, the results of this study showed statistical significance for some symptoms, which confirmed the results obtained from previous studies indicating that these symptoms are characteristic symptoms of HB.^3^,^4^ Another limitation is that this study only investigated the difference between HB and depression. Further studies are needed to compare HB to anxiety or somatization disorders.

Patients with anger syndrome similar to HB may be found in other cultures as well. In the USA, similar conditions include intermittent explosive disorder and anger attacks in patients with major depressive disorder or panic disorder.^19^ Intermittent explosive disorder involves serious assaults or destruction of property, with an early age at onset.^18^ However, anger in HB is mainly a subjective and chronically persistent anger, which is partially suppressed or intermittently experienced. Also, HB is frequently found in middle-aged or older women. However, some somatic symptoms reported in anger attacks including hot flashes, feeling hot, face getting red, chest tightness and palpitation^19^ are common in HB. As 5 of patients with self-labeled HB were not diagnosed as having any DSM-IV disorder, it is possible that there exist patients who have only HB (anger syndrome) as a disorder. We are currently investigating this hypothesis with a larger number of subjects. Moreover, in other cultures including the USA, patients may visit psychiatrists with anger-related problems, such as anger attacks without a major depressive disorder or a mild form of intermittent explosive anger without serious acts of assault or destruction of property.

However, considering the dynamic relationship between anger and depression,^20^ the concept of mixed anxiety-depression,^21^ frequently comorbidity of intermittent explosive disorder with mood and anxiety disorders,^17^ frequent comorbidity and mixed forms between anger, anxiety, and depressive disorders may be highly possible. Also, in this study, some HB symptoms are correlated with depressed mood or anxiety. Theoretically, anger related to precipitating stress may be suppressed or transformed during the chronic course, according to the individual's predisposition and selective adoption of defense mechanisms and coping strategies, into an anger syndrome, anxiety state, depressive syndrome, somatization disorder, or even a paranoid or manic state, with depressive disorders probably being the most common manifestation. Even patients with intermittent explosive disorder, who have to or can suppress violent expressions of anger, may later develop a chronic anger syndrome like HB.

However, anger has been described as a "forgotten emotion" in psychiatric research.^22^ Nevertheless, American clinical psychologists have conceptualized clinical models of problematic anger and have suggested cognitive-behavioral treatment for anger patients.^23^ Why has "anger disorder" developed in Korea but not in Western countries? The difference may be cultural^24^ and an understanding of these cultural differences requires future cross-cultural research. International comparative studies are necessary to acquire evidence of this anger syndrome, including its identification, clinical correlations, bio-psycho-social basis and treatment.

## Figures and Tables

**TABLE 1:**
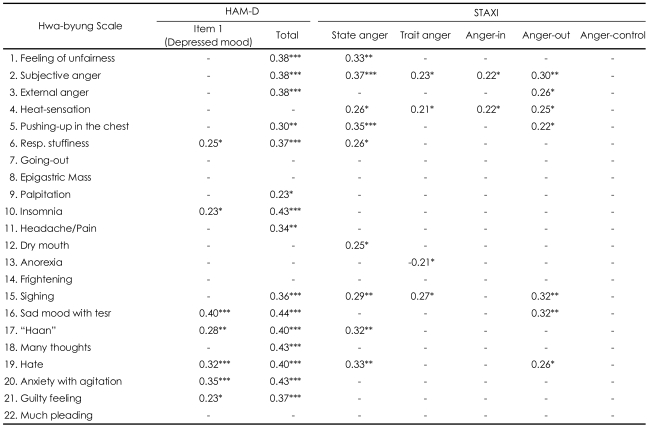
Correlation coefficient (r) between hwa-byung symptoms and HAM-D and STAXI

^*^p<0.05, ^**^p<0.01, ^***^p<0.001. "Haan": a Korean culture-related depressed sentiment related to hard life, HAM-D: Hamilton's rating scale for depression, STAXI: State and Trait Anger Inventory

**TABLE 2:**
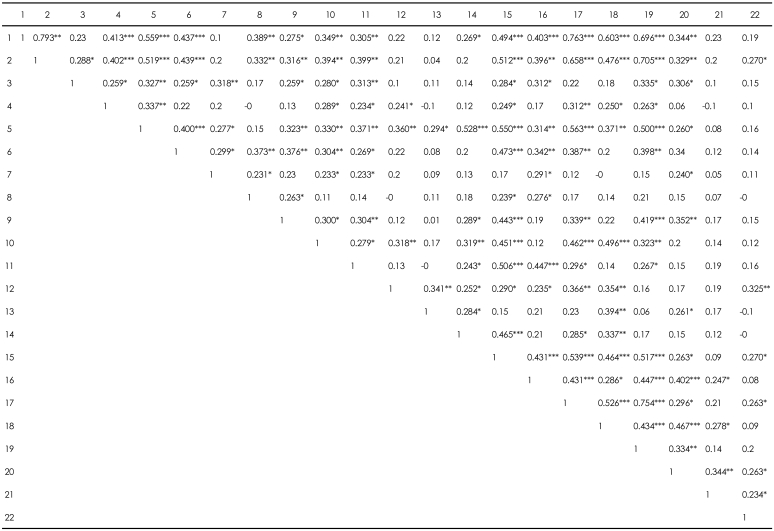
Correlation coefficient (r) in correlation matrix among hwa-byung symptoms

1: feeling of unfairness, 2: subjective anger, 3: external anger, 4: heat sensation, 5: pushing-up, 6: respiratory stuffiness, 7: going, out, 8: epigastric mass, 9: palpitation, 10: insomnia, 11: headache, 12: dry mouth, 13: anorexia, 14: easily frightening, 15: sigh, 16: sad mood with tear, 17: haan, 18: many thoughts, 19: hate, 20: anxiety with agitation, 21: guilty feeling, 22: much pleading. ^*^p<0.05, ^**^p<0.01, ^***^p<0.001
